# COVID-19-associated fungal infections in Iran: A systematic review

**DOI:** 10.1371/journal.pone.0271333

**Published:** 2022-07-11

**Authors:** Tina Nazari, Fatemeh Sadeghi, Alireza Izadi, Setayesh Sameni, Shahram Mahmoudi

**Affiliations:** 1 Department of Medical Geriatrics, School of Medicine, Tehran University of Medical Sciences, Tehran, Iran; 2 Department of Parasitology and Mycology, School of Medicine, Iran University of Medical Sciences, Tehran, Iran; 3 Department of Medical Parasitology and Mycology, School of Medicine, Kerman University of Medical Sciences, Kerman, Iran; 4 Medical Mycology and Bacteriology Research Center, Kerman University of Medical Sciences, Kerman, Iran; 5 Department of Medical Sciences, Shahrood Branch, Islamic Azad University, Shahrood, Iran; Gulu University, UGANDA

## Abstract

**Objectives:**

This systematic review aims to summarize the mycological and clinical features of COVID-19-associated fungal infections (CAFIs) in Iran.

**Methods:**

PubMed, Web of Science, Scopus, Cochrane Library, SID, Magiran, IranDoc, and Google Scholar were searched for Persian and English articles published from January 1, 2020, to November 5, 2021, using a systematic search strategy. Studies on Iranian patients suffering from CAFIs were included in the review.

**Results:**

Twenty-two studies comprising 169 patients were retrieved. Reported CAFIs included candidiasis (85, 50.30%), mucormycosis (35, 20.71%), aspergillosis (29, 17.16%), fusariosis (6, 3.55%), three cases caused by rare pathogens *(Rhodotorula mucilaginosa*, *Diaporthe foeniculina*, and *Sarocladium kiliense*) and 11 (6.51%) uncharacterized mold infections. The most common underlying diseases were diabetes (67/168, 39.88%), cardiovascular diseases (55/168, 32.74%), and hypertension (43/168, 25.59%). The use of antibiotics (111/124, 89.52%), corticosteroids (93/132, 70.44%), and mechanical ventilation (66, 51.16%) were the most common predisposing factors. Totally, 72 (50.35%) of 143 patients with CAFIs died (data were not available for 26 patients).

**Conclusion:**

Fungal infections are evident to be a complication of COVID-19 in Iran; thus, clinicians should consider them as a differential diagnosis, especially in patients with comorbidities and previous antibiotic or corticosteroid use.

## Introduction

The coronavirus disease 2019 (COVID-19), which started as a pneumonia epidemic in Wuhan, China, in December 2019, has become a global issue due to its high prevalence and rapid transmission [1]. It has been diagnosed in hundreds of million cases and killed millions of people around the world [2]. In addition to the harmful effects of the virus, such as dysregulation of immune response and direct damage to pulmonary and extra-pulmonary tissues, COVID-19 can be accompanied by infections caused by other microorganisms [3, 4].

Fungal, bacterial, and viral co-infections and super-infections have been detected in patients infected with severe acute respiratory syndrome coronavirus 2 (SARS-CoV-2), leading to more difficult management and increased morbidity and mortality [5]. Fungal infections have been reported in 3.7% of COVID-19 patients, with a higher occurrence (9.6%) in ICU-admitted cases [6]. They have been shown to increase fatality in COVID-19 patients, which can be reduced with the prescription of antifungal medications [7]. Aspergillosis, candidiasis, and mucormycosis are the most commonly reported CAFIs while other infections such as cryptococcosis, *Pneumocystis jirovecii* pneumonia, coccidioidomycosis, Paracoccidioidomycosis, and histoplasmosis have also been disclosed [8, 9].

COVID-19 weakens the cellular immunity due to decreasing T lymphocytes and alters the respiratory and gastrointestinal microbiota, which can make patients more susceptible to fungal infections [10, 11]. Long stays at hospitals, especially ICU, mechanical ventilation, the use of broad-spectrum antibiotics and corticosteroids in COVID-19 patients may also contribute to an increased risk of mycoses [12, 13].

Following the COVID-19 spread in Iran, cases of CAFIs have been reported from different parts of the country [14–16]. To date, there is no comprehensive study of fungal infections in patients with COVID-19 in Iran. In this systematic review, we aim to summarize the studies that have reported CAFIs in Iran.

## Methods

This systematic review was conducted in accordance with the Preferred Reporting Items for Systematic Reviews and Meta-Analyses (PRISMA) (S1 File) [17], and the protocol of this review was registered in the International Prospective Register of Systematic Reviews (PROSPERO registration code: CRD42021287258). We performed a comprehensive search for studies concerning fungal infections diagnosed in COVID-19 patients reported from Iran during 2020 and 2021.

Electronic databases, including PubMed, Web of Science, Scopus, Cochrane Library, SID, Magiran, and IranDoc, as well as Google Scholar search engine, were searched on November 5, 2021. The systematic search was conducted with a combination of keywords such as ‘COVID-19’, ‘SARS-CoV-2’, ‘fungal infections’, ‘candidiasis’, ‘mucormycosis’, ‘aspergillosis’, and ‘Iran’. An exhaustive list of keywords and MeSH terms used for PubMed search is provided in S2 File. The search results were filtered for Persian and English languages and publication date from January 1, 2020. There was no limitation on the study design. Moreover, references of selected articles were investigated for additional relevant articles.

The inclusion criteria were: (1) the studies concerned patients diagnosed with COVID-19, (2) the subjects became infected with a type of fungi simultaneously or after contracting COVID-19, (3) the studies took place in Iran, (4) the articles were written in English or Persian. Reviews, letters, animal studies, and studies that did not contain clinical data of infected patients were excluded.

Titles and abstracts of the retrieved articles were screened independently by two reviewers. Next, the full-text articles were reviewed by two independent authors to determine the eligibility of reports based on the inclusion criteria. The risk of bias of the studies was assessed by two independent reviewers using the Joanna Briggs Institute critical appraisal tools (https://jbi.global/critical-appraisal-tools). In case of any disagreement between the two authors in each stage, a third author was consulted. Two researchers independently extracted data, including bibliographic, demographic, and clinical details. Disagreement between data collectors was resolved through discussing a third reviewer.

The bibliographic data included the first author, year of publication, and study design. Demographic features consisted of the province, sample size, incidence, mortality rate, age, gender, past medical history, and drug history. Clinical details included signs and symptoms, laboratory and radiological findings, diagnostic methods, and treatment separately for COVID-19 and fungal infections; as well as duration between COVID-19 and fungal infection diagnosis, genera and species of fungi, site of fungal infection, antifungal susceptibility tests, and outcome of treatment. The extracted data were reviewed, and variables with an acceptable proportion of available values were summarized as mean ± SD or frequency (percentage).

## Results

**Fig 1:**
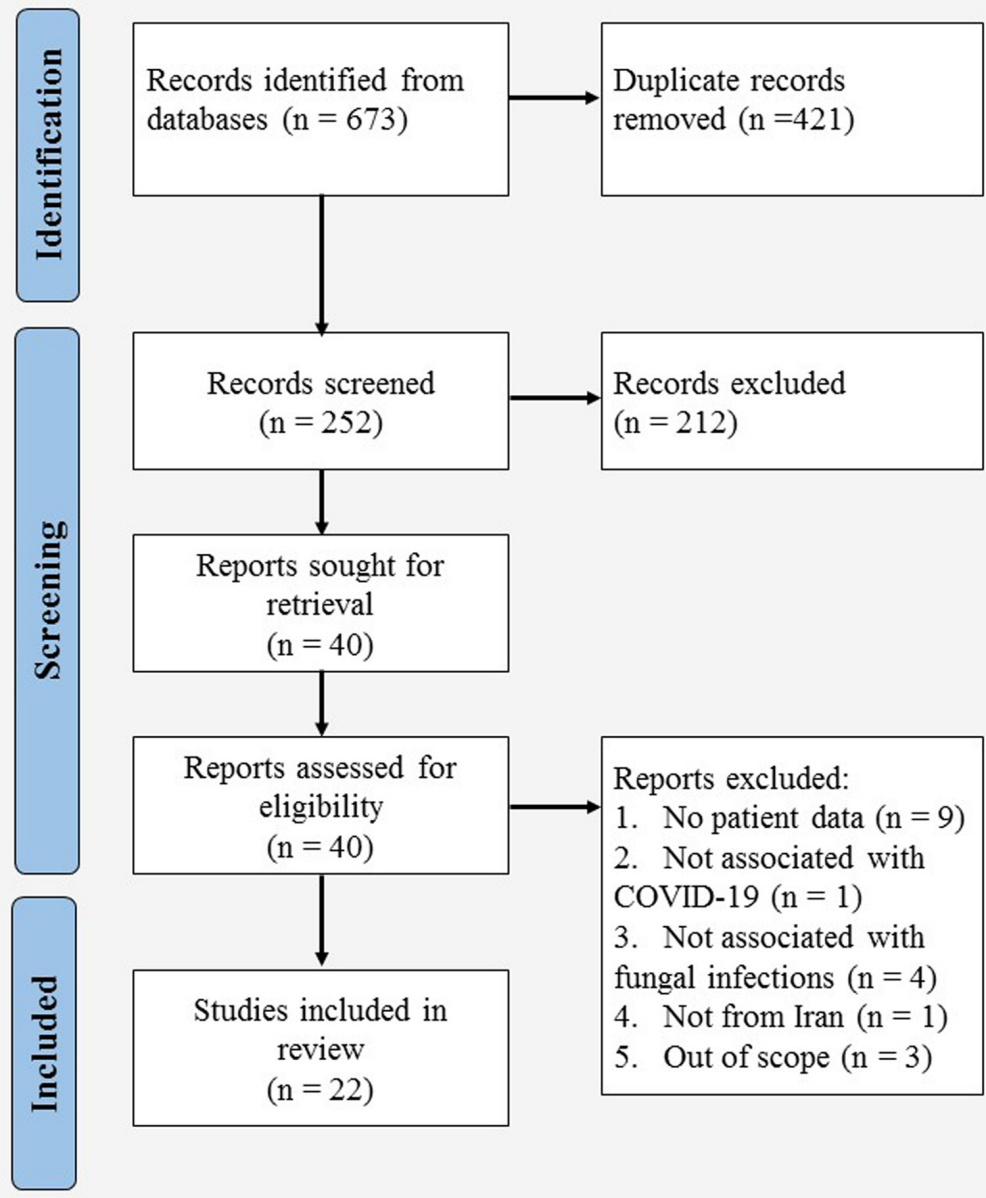
The PRISMA flow diagram for selection of studies reporting data on COVID-19-associated fungal infections in Iran up to November 5, 2021.

Database searching resulted in 673 articles, of which 22, including 16 case reports and six cross-sectional studies, were included in the present study (Fig 1) [15, 16, 18–37]. Details of the articles and results of the quality assessment are presented in Table 1 and S3 File, respectively. The reviewed studies comprised 169 cases of fungal infections among Iranian COVID-19 patients with the dominance of candidiasis (85, 50.30%), followed by mucormycosis (35, 20.71%), aspergillosis (29, 17.16%), and fusariosis (6, 3.55%). Three cases of rare infections and 11 (6.51%) cases of uncharacterized mold infections were also found. Details of comorbidities, risk factors, diagnosis, treatment, and outcome are summarized in Tables 2 and 3, separately for the three common fungal infections.

**Table 1:** Details of 22 studies with data of COVID-19-associated fungal infections among Iranian patients published up to November 5, 2021.

First author	Year of publication	Type of study	Type of fungal infection (N of patients)	Province	Ref.
Abolghasemi	2021	Case report	Aspergillosis (1)	Tehran	[18]
Arastehfar	2021	Cross-sectional	Candidemia (6), *Rhodotorula mucilaginosa* fungemia (1)	Khorasan Razavi	[19]
Davoodi	2021	Case report	Candidemia and endocarditis (1)	Mazandaran	[20]
Fazeli	2021	Cross-sectional	Mucormycosis (12)	Kermanshah	[21]
Ghazanfari	2021	Cross-sectional	Aspergillosis (22), fusariosis (6), unidentified mold infections (11), *Diaporthe foeniculina* infection (1)	Mazandaran	[16]
Hakimifard	2021	Case report	Aspergillosis (1)	Isfahan	[22]
Heydarifard	2021	Case report	Mucormycosis (1)	Tehran	[23]
Hosseinikargar	2021	Case report	Aspergillosis (1)	Khorasan Razavi	[24]
Karimi-Galougahi	2021	Case report	Mucormycosis (1)	Tehran	[25]
Khodavaisy	2021	Case report	Aspergillosis (1)	Tehran	[26]
Mehrabi	2021	Case report	Mucormycosis (1)	Bushehr/Fars	[27]
Mohammadi	2021	Case report	Mucormycosis (1)	Qazvin	[28]
Nasri	2020	Case report	Aspergillosis (1)	Isfahan	[29]
Ostovan	2021	Case report	Mucormycosis (1)	Fars	[30]
Pakdel	2021	Cross-sectional	Mucormycosis (15)	Tehran	[15]
Ranjbar-Mobarake	2021	Case report	*Sarocladium kiliense* infection (1)	Isfahan	[31]
Salehi	2020	Cross-sectional	Oropharyngeal candidiasis (53)	Tehran	[32]
Salehi	2021	Case report	Aspergillosis (1)	Tehran	[33]
Sharifpour	2021	Case report	Aspergillosis (1)	Mazandaran	[34]
Shirvani	2021	Cross-sectional	Pulmonary candidiasis (25)	Tehran	[35]
Tabarsi	2021	Case report	Mucormycosis (1)	Tehran	[36]
Veisi	2021	Case series	Mucormycosis (2)	Tehran	[37]

**Table 2:** Frequency of comorbidities and risk factors in COVID-19-associated fungal infection cases reported from Iran up to November 5, 2021.

Variables	Frequencies based on the type of fungal infection		
	Candidiasis	Mucormycosis	Aspergillosis
**Past medical history**	Diabetes mellitus (21, 24.70%)	Diabetes mellitus (27, 77.14%)	Diabetes mellitus (10, 35.71%)
	Immunosuppressive diseases (25, 29.41%)	Hypertension (17, 48.57%)	Hypertension (13, 46.43%)
	Cardiovascular diseases (29, 34.11%)	Cardiovascular disease (7, 20%)	Cardiovascular disease (11, 39.28%)
	Pulmonary diseases (5, 5.88%)	Pulmonary diseases (4, 11.43%)	Pulmonary diseases (5, 17.24%)
	Malignancy (8, 9.41%)	Malignancy (2, 5.71%)	Malignancy (4, 14.28%)
	Chronic kidney disease (11, 12.94%)	Chronic kidney disease (2, 5.71%)	Chronic kidney disease (2, 7.14%)
	Others (23, 27.05%)	Others (9, %)	Others (4, 13.79%)
	Healthy (5, 5.88%)	Healthy (4, 11.43%)	Healthy (3, 10.71%)
			Unknown (1)
**Mechanical ventilation**	22/60, 36.7%	3/22, 13.64%	23/27, 85.19%
	Unknown (25)	Unknown (13)	Unknown (2)
**Central venous catheter**	11/60, 18.3%		
	Unknown (25)	Unknown (35)	Unknown (29)
**Intensive care unit hospitalization**	33/60, 55%	9/26, 34.62%	26/27, 96.3%
	Unknown (25)	Unknown (9)	Unknown (2)
**Corticosteroid therapy**	25/54, 46.3%	23/32, 71.87%	26/27, 96.3%
	Unknown (31)	Unknown (3)	Unknown (2)
**Antibiotic consumption**	56/60, 93.33%	10/16, 62.5%	25/28, 89.29%
	Unknown (25)	Unknown (19)	Unknown (1)

**Table 3:** Description of diagnosis, treatment and outcome of COVID-19-associated fungal infection cases reported from Iran up to November 5, 2021.

Variables	Frequencies based on the type of fungal infection		
	Candidiasis	Mucormycosis	Aspergillosis
**COVID-19 diagnostic method (n, %)**	PCR (29, 48.33%)	PCR (18, 51.43%)	PCR + CT (28, 96.55%)
	PCR + CT (1, 1.66%)	PCR + CT (14, 40%)	CT (1, 3.45%)
	Clinical (30, 50%)	CT (2, 5.71%)	
	Unknown (25)	Clinical (1, 2.86%)	
**Days between COVID-19 and fungal infection diagnosis (mean ± SD)**	8.97 ± 8.89	17.18 ± 12.44	29 ± 19.3
	Unknown (25)	Unknown (1)	Unknown (23)
**Fungal infection diagnostic method (n, %)**	Microscopic + molecular + culture (53, 62.35%)	Microscopic + imaging (18, 51.43%)	Culture + biomarker^a^ (17, 58.62%)
	Molecular (25, 29.41%)	Microscopic (15, 42.86%)	Culture (5, 17.24%)
	Molecular + culture (6, 7.06%)	Microscopic + culture + molecular + imaging (2, 5.71%)	Biomarker + imaging (2, 6.89%)
	Molecular + culture + imaging (1, 1.17%)		Microscopic + culture + Molecular + biomarker + imaging (2, 6.89%)
			Microscopic + culture + Molecular + imaging (2, 6.89%)
			Microscopic + culture + imaging (1, 3.45%)
**Fungal infection treatment (n, %)**	FCZ (22, 36.66%)	AMB (1, 2.86%)	VCZ (2, 6.9%)
	FCZ + NYS (20, 33.33%)	AMB + SI (25, 71.43%)	VCZ + CSP (1, 3.45%)
	NYS (10, 16.66%)	AMB + PSZ+ SI (5, 14.28%)	VCZ + AMB (2, 6.9%)
	FCZ + CSP (5, 8.33%)	AMB + PSZ + CSP + SI (1, 2.86%)	FCZ + AMB + surgical intervention (1, 3.45%)
	CSP (1, 1.66%)	AMB + CSP + SI (2, 5.71%)	AMB (1, 3.45%)
	AMB (1, 1.66%)	Systemic antifungal + SI (1, 2.86%)	No treatment (22, 75.86%)
	No treatment (1, 1.66%)		
**Case fatality rate (%)**	16.66%	55.88%	86.2%
	Unknown (25)	Unknown (1)	

PCR: Polymerase chain reaction; CT: computerized tomography scan; FCZ: fluconazole; AMB: Amphotericin B; VCZ: voriconazole; NYS: nystatin; CSP: caspofungin; PSZ: posaconazole; SI: surgical intervention.

^a^Either galactomannan or β-D-glucan in serum or bronchoalveolar lavage sample.

### Candidiasis among Iranian COVID-19 patients

Four studies comprising 85 cases of candidiasis were found among Iranian COVID-19 patients. The mean ± SD of age was 61.98 ± 17.10 years, and 44 (51.76%) patients were female. The cases included oropharyngeal candidiasis (53, 62.35%), pulmonary candidiasis (25, 29.41%), candidemia (6, 7.06%), and endocarditis (1, 1.18%). In total, 97 *Candida* isolates were recovered from the patients (9 cases of mixed infection), and *C*. *albicans* (74, 76.29%) was the most common species, followed by *C*. *glabrata* (4, 4.71%), *C*. *dubliniensis* (3, 3.53%), *C*. *tropicalis* (2, 2.35%), *C*. *parapsilosis* (1, 1.18%), and *C*. *krusei* (1, 1.18%).

Mixed infection cases included *C*. *albicans + C*. *glabrata* (2, 2.35%), *C*. *albicans + C*. *parapsilosis* (2, 2.35%), *C*. *albicans + C*. *tropicalis* (1, 1.18%), *C*. *albicans + C*. *dubliniensis* (1, 1.18%), *C*. *albicans + C*. *glabrata + C*. *dubliniensis* (2, 2.35%), and *C*. *albicans + C*. *glabrata + C*. *parapsilosis* (1, 1.18%).

### Mucormycosis among Iranian COVID-19 patients

Nine studies composed of 35 cases of mucormycosis, 19 (54.29%) males and 16 (45.71%) females, were retrieved. The mean ± SD of age was 55.97 ± 12.93 years. All patients had rhino-orbital-cerebral manifestations, with orbital involvement in 32 (91.43%) and rhino-sinusitis in three (8.57%) cases. The orbital infections comprised of rhino-sino-orbital (17, 48.57%), rhino-orbital (7, 20%), sino-orbital (5, 14.29%), orbital (2, 5.71%), and rhino-sino-orbito-cerebral (1, 2.86%) involvements. Definitive identification of the causative agents was made for only two cases of *Rhizopus arrhizus* (*R*. *oryzae*).

### Aspergillosis among Iranian COVID-19 patients

Eight studies comprising 29 cases of aspergillosis were reported among patients with COVID-19 from Iran. The patients were 62.97 ± 12.68 years old, and 15 (51.72%) were male. Pulmonary infection (27, 93.1%) was the most common clinical form of the disease; in addition, one patient (3.45%) with disseminated (pulmonary + cerebral) and one patient (3.45%) with rhinosinusitis were reported. In total, 26 out of 29 isolates were identified to the species level. *Aspergillus flavus* (15/26, 57.69%) was the most common etiological agent, and the remaining isolates included *A*. *fumigatus* (3, 11.54%), *A*. *japonicus* (3, 11.54%), *A*. *niger* (2, 7.69%), *A*. *ochraceus* (1, 3.85%), *A*. *terreus* (1, 3.85%), and *A*. *tubingensis* (1, 3.85%).

### Fusariosis among Iranian COVID-19 patients

Six cases of pulmonary fusariosis, two males (33.33%) and four females (66.67%), were found in PCR-confirmed COVID-19 cases with a mean ± SD age of 67 ± 12.66 years. The causative agents, including *Fusarium incranatum* (3, 50%), *F*. *fujikuroi* (1, 16.67%), *F*. *equiseti* (1, 16.67%), and *F*. *solani* (1, 16.67%) were detected through bronchoalveolar lavage (BAL) culture; and five patients had positive BAL galactomannan (GM) test.

Hypertension (4, 66.67%), cardiovascular diseases (3, 50%), diabetes mellitus (2, 33.33%), and chronic kidney diseases (1, 16.67%) were the reported underlying diseases. All six patients were admitted to ICU, mechanically ventilated, and received antibiotics and corticosteroids. None of the patients received antifungal medications, and all of them regrettably died.

### Rare or uncharacterized fungal infections among Iranian COVID-19 patients

Three cases of CAFI caused by rare fungi were reported:

1. Fungemia due to *Rhodotorula mucilaginosa* in an ICU-admitted, mechanically ventilated, 68-year-old female without underlying diseases, who was effectively treated with fluconazole.
2. Disseminated infection due to *Sarocladium kiliense* in a 74-year-old diabetic female receiving corticosteroid and antibiotics. She did not survive despite receiving liposomal amphotericin B.
3. Pulmonary infection due to *Diaporthe foeniculina* in a 67-year-old female with diabetes, hypertension, chronic kidney disease and ischemic heart disease, with a history of ICU admission, mechanical ventilation, corticosteroid and antibiotic therapy. The patient received no antifungal treatment and died.

Eleven cases of uncharacterized pulmonary mold infections were also found in ICU-admitted patients who underwent mechanical ventilation, corticosteroid and antibiotic therapy. They were detected by high levels of GM (serum GM index > 0.5 or BAL GM index ≥ 1). None of the patients were prescribed antifungals, and all except one passed away.

## Discussion

This systematic review showed that the most common fungal infections reported in Iranian COVID-19 patients are candidiasis, mucormycosis, and aspergillosis. Previous global reviews also found members of *Candida*, *Aspergillus*, and Mucorales among the most frequent fungi causing secondary infections in people with COVID-19 [6, 8, 9, 38].

Results of the present review are based on a limited number of studies, mainly case reports, which are prone to variation by future reports. Meanwhile, the thorough systematic search and exhaustive data collection provide a comprehensive description of CAFIs to the date of database searching across the country, which could be considered a strength of this report.

### COVID-19-associated candidiasis (CAC)

*Candida* is the most prevalent fungal pathogen causing superinfections and the second most common source of coinfections in COVID-19 patients [39]. A cross-sectional study of the patients’ registry in an American hospital showed that 12% of patients diagnosed with COVID-19 had candidiasis, and patients with candidiasis were 3.73 times more likely to be diagnosed with COVID-19 [40]. In this review, around half of the reported CAFI cases were caused by *Candida* spp., the most common fungal pathogens in Iranian COVID-19 patients.

The exact pathogenesis of CAC is not clarified, but several factors have been suggested to explain the predisposition of patients with COVID-19 to *Candida* infections. The SARS-CoV-2 infection can cause lymphopenia and consequent impairment of immune defense against fungal agents including *Candida* spp. [41, 42]. Severe COVID-19 is associated with elevation of blood lactate and acidosis [43, 44], which enables *Candida* to restructure its cell wall to mask β-glucans and escape the recognition by the host immune system [45]. Moreover, an important virulence factor of *Candida* that helps it evade phagocytosis is the ability to form biofilms, which can be triggered by COVID-19 through increasing oxidative stress and pH imbalance [41].

On the other hand, COVID-19 patients are at an increased risk of contracting candidiasis because of the treatments they receive. Consumption of corticosteroids, antibiotics, and TNF-α inhibitors, as well as undergoing mechanical ventilation, central venous catheterization, and extracorporeal membrane oxygenation (ECMO) are risk factors for developing candidiasis [41, 46–48]. Concordant to previous studies, a significant proportion of the patients in our review were exposed to antibiotic consumption, corticosteroid use, mechanical ventilation, and central venous catheterization. However, we did not find any report of patients with CAC who had received a TNF-α inhibitor or went through ECMO, as these are less commonly indicated for COVID-19 treatment.

Another predictor of developing CAC is admission to ICU, which is often accompanied by interventions such as mechanical ventilation and central venous catheterization and can raise the risk of nosocomial *Candida* infections [41]. In our study, 55% of the CAC cases (100% of patients with candidemia) stayed in ICU, emphasizing the need for more attention to ICU patients in terms of prompt diagnosis of candidiasis.

A global review showed that CAC is more likely to involve male patients with more than 40 years of age [38]. This is probably because of the fact that higher age and male gender are associated with more severe COVID-19 and the need for ICU admission [49, 50]. Expectedly, the Iranian CAC cases were about 62 years old on average. However, with female patients being reported slightly more than males, no significant gender difference was found in our review.

The most common species causing CAC throughout the world is *C*. *albicans*, which has also been the most frequent causative agent of candidiasis before the pandemic [38, 51]. Similarly, the majority of *Candida* isolates obtained from Iranian patients were identified as *C*. *albicans*. The following frequent pathogens in Iran were *C*. *glabrata*, *C*. *dubliniensis*, *C*. *parapsilosis*, and *C*. *tropicalis*; which were also among the most common species in other countries [52–56]. Furthermore, Salehi et al. reported one case of CAC caused by *C*. *krusei* [32]. A less prevalent species, this pathogen was detected in two other CAC cases from India and Spain [54, 55].

While cases of *C*. *auris*, *C*. *lusitaniae*, *C*. *orthopsilosis*, and *C*. *inconspicua* were reported globally, they were not found in Iranian patients [55, 57–62]. This is not surprising as we know there are regional differences in the global distribution of *Candida* species [63]. For example, while *C*. *auris* outbreaks have been reported from India, Pakistan, USA, Venezuela, South Africa, Spain and the United Kingdom [64]; only limited sporadic cases of this species were reported from Iran [65–67]. In addition, *C*. *auris* and other rare pathogens might have been missed due to the lack of proper diagnostic tools in Iran; thus, the differences might result from the use of culture and PCR for identifying the causative agents [65, 68, 69].

Oropharyngeal candidiasis (OPC) is a common form of *Candida* infection, which has been reported in people with COVID-19 as well [48, 70]. In the majority of CAC patients described in this review, the infection was localized in the oropharyngeal area. Pulmonary candidiasis rarely happens in critically ill patients with multiple risk factors [71, 72]. Although Shirvani et al. reported 25 cases of COVID-19 patients with *Candida* spp. detected in their BAL fluid [35], no other pulmonary CAC was reported from other countries. There is no consensus on whether *Candida* can actually cause pulmonary infection or whether its presence in BAL samples is due to colonization [73, 74].

Candidemia, which is 3–8 times more frequent in COVID-19 patients compared to the non-COVID-19 population [75, 76], was confirmed in six Iranian COVID-19 patients [19]. Moreover, a case of endocarditis caused by *C*. *tropicalis* was reported in an Iranian patient with prosthetic heart valves, who died despite receiving liposomal amphotericin B [20]. Another case of endocarditis caused by *C*. *albicans* was diagnosed in an Italian COVID-19 patient with a central venous catheter [77]. Fungal endocarditis is a rare and hard-to-treat condition leading to a 50% mortality, with *C*. *albicans* being the causative agent in 24–46% of cases [78]. In a study conducted in 2009–2011 on Iranian patients with infective endocarditis who did not respond to antibacterial treatment, *C*. *albicans* has been documented in two out of eleven cases. These two patients were intravenous drug abusers and responded to treatment with surgical removal and amphotericin B [79]. What this study brings to mind is that surgical removal of the vegetations might have improved the outcome as the *C*. *tropicalis* isolate was proved to be sensitive to amphotericin. Based on a review of global studies, CAC has a mortality rate ranging from 11 to 100% based on the infection site and clinical conditions [38]. In this study, all cases with candidemia and endocarditis died; nonetheless, the overall mortality rate was 16.66% because of the low fatality of OPC. Studies with large sample sizes and adjustments of underlying factors are warranted to estimate the attributable mortality of different categories of CAC.

### COVID-19-associated mucormycosis (CAM)

Over the last decade, mucormycosis has been more frequently diagnosed, especially in India [80]. A pre-pandemic study showed an increased incidence (about 2.5 times) of mucormycosis during the years 2008–2014 in Iran [81]. Besides, this infection has become 50 times more prevalent in the COVID-19 era globally [82]. CAM has been primarily seen in India, while cases from other countries, including Iran, have also been reported [83].

Two major risk factors of CAM are diabetes mellitus (present in 79–85% of patients) and use of corticosteroids (prescribed in 76.3–85% of cases) [8, 38, 84–86]. Similarly, the most frequent risk factor in Iranian CAM patients was diabetes mellitus, present in the majority (77.14%) of patients, and corticosteroid treatment with a 71.87% frequency. It should be noted that corticosteroids are not always prescribed correctly, as it is demonstrated that only 28.8% of cases in India and 52.6% in other countries received corticosteroids based on indication and with accurate dosage [83]. Male gender might also be related to a higher incidence of CAM, as several studies have shown more than 70% of patients were men [38, 85–88]. However, with men accounting for 54% of Iranian patients, the male preponderance was not as significant as in previous studies. Since limited cases have been studied, more extensive research is needed to understand the gender distribution of CAM both in Iran and the world. The average age of CAM patients was 56 years in Iran, roughly similar to a 55 years median in worldwide studies [89].

Making up 57% of culture results, *Rhizopus* spp. are the most commonly identified fungi in CAM cases worldwide [84]. *Mucor* spp. and *Lichteimia* spp. were also diagnosed in a small number of cases [38]. In the studies of this review, the species were not identified except for two cases of *R*. *arrhizus*. The rationale for the lack of data about species is that identifying mucormycosis agents to the level of genera and species for clinical management is not strongly supported by evidence, although recommended for epidemiological purposes [90]. Besides, culture has a low sensitivity (50%) to identify the species, and molecular techniques are not yet standardized and yield heterogeneous results [91].

The most common form of the disease, with or without COVID-19, is rhino-orbital-cerebral mucormycosis (ROCM) [80, 92]. All 35 CAM cases from Iran fell into this category, with a 91.43% orbital and 2.86% cerebral involvement. While not found in our review, several cases of lung, bone, gastrointestinal, and skin involvement were reported from other countries [93–101]. The absence of reported CAM cases of organs other than the nose, sinus, and orbits from Iran might be because of the rarity of these infections, the non-specificity of their presentations, and the lack of awareness among clinicians about the uncommon presentations of mucormycosis. The mortality rate of CAM has been estimated at 30–52% in various review studies [38, 84, 86, 102]. In the present review, 55.88% of patients died despite treatment, comparable to the 61.9% mortality rate in countries other than India [83]. The mortality rate of CAM in India is lower (14–36.5%), probably because of the higher prevalence of ROCM in Indian patients [83, 87], as it is known that disseminated and pulmonary mucormycosis are associated with higher mortality rates than ROCM and cutaneous infection [90]. Nevertheless, since none of the Iranian cases had disseminated or pulmonary infection, this cannot be the reason for the higher fatality rate. However, the higher prevalence of orbital involvement in the Iranian (91.43%) compared to the Indian (72%) CAM cases might be a sign of neglect and delayed diagnosis, as well as the possible source of the poorer prognosis, as orbital involvement with vision loss is associated with a higher mortality rate [87].

The treatment of choice for mucormycosis is amphotericin B, which is more effective when combined with surgical interventions [103]. Isavuconazole and posaconazole, alone or in combination with amphotericin B, are recommended as first-line or salvage therapy [104, 105]. Almost all patients in our review received amphotericin B and surgical interventions (Table 3). Although we did not analyze the data statistically, it was noticed that all eight cases that received one or two drugs (caspofungin, posaconazole or both) in addition to Amphotericin B survived while monotherapy with Amphotericin B led to ~73% mortality. This highlights the need for further clinical trials as the efficiency of combination therapy is proposed by limited studies yet not supported by enough evidence [104, 106].

### COVID-19-associated aspergillosis (CAA)

Observed in 7.7–27.7% of patients with severe COVID-19, aspergillosis is another fungal infection frequently seen along with this viral infection [107]. COVID-19-associated pulmonary aspergillosis (CAPA) affects up to 35% of ICU patients with COVID-19, leading to a 54.9% mortality rate [108, 109]. CAA of the nose and sinuses was also reported from India and Egypt [110–113]. Twenty-nine CAA cases were reported from Iran, of which 27 were diagnosed with CAPA, in addition to two patients with rhinosinusitis and disseminated infection.

CAPA is almost exclusive to ICU-admitted patients, while *Aspergillus* rhinosinusitis was reported in several outpatient COVID-19 cases [108, 111, 114, 115]. All except one of the Iranian cases with available data were admitted to ICUs. Corticosteroids (96.3%), antibiotics (89.29%), and mechanical ventilation (85.19%) were used in a large proportion of the patients in our review. A global study revealed a high frequency of mechanical ventilation (94.1%), but corticosteroid use was less frequent (52.7%) [115]. The exact antibiotic use frequency has not been reported in CAPA patients, but a 75% proportion could be presumed based on data from ICU-admitted COVID-19 patients [116]. The differences can be explained by the prescription of dexamethasone, ceftriaxone and azithromycin per institutional protocol in all patients of the Ghazanfari et al. study, which is the reference for the majority of our patients [16].

Hypertension was the most common comorbidity both in our study (46.43%) and a worldwide review (24%) [108]. Another study reported cardiovascular diseases (50.5%) as the most prevalent underlying condition in CAPA patients [115], and we found it in 39.28% of patients in our review. Diabetes mellitus, malignancies and pulmonary diseases are also common among CAPA patients both in Iran and other countries [108, 115, 117]. However, none of these comorbidities are significantly different in CAPA and other ICU-admitted COVID-19 patients [117].

The most prevalent causative agent of CAPA is *A*. *fumigatus*, observed in more than 80% of cases globally [108, 114, 116]. However, in Iran, *A*. *fumigatus* was the second most common pathogen after *A*. *flavus*, identified in more than half of Iranian CAPA cases. This is not unexpected as previous studies on species distribution of *Aspergillus* in Iran before the COVID-19 outbreak showed that *A*. *flavus* had the highest prevalence, followed by *A*. *fumigatus*, *A*. *niger*, and *A*. *terreus* [118, 119]. The disseminated aspergillosis case was caused by *A*. *ochraceus*, which has not been reported, to our knowledge, in other COVID-19 patients. *A*. *ochraceus* is a rare pathogen, previously described in two case reports of chronic pulmonary aspergillosis and allergic bronchopulmonary aspergillosis [120, 121].

The fatality rate of CAA in Iran was estimated at 86.2%, which is remarkably higher than CAPA all-cause mortality rates in global studies (42.6–54.1%) [108, 115, 122]. The global rate is even lower when the cause of death is limited to CAPA (17.2%) [115]. Apostolopoulou et al. found an association between age, male sex and pulmonary diseases with mortality of CAPA in a global review [108]. None of these factors can be the reason for the higher mortality in the Iranian patients, who were on average 62.97 years old (vs 67% worldwide), 51.72% male (vs 75.4% worldwide), and 17.85% with pulmonary comorbidities (vs 20% worldwide) [108]. The difference in the mortality rate of Iranian and global cases can be explained by the fact that 22 out of 29 patients, for an unknown reason, did not receive antifungal treatment [16]. Only two out of seven patients who received antifungal treatment survived; both received voriconazole, which is considered the first-line therapy of invasive pulmonary aspergillosis and is the most frequently prescribed drug for CAPA worldwide [108, 114, 116].

### COVID-19-associated fusariosis and other CAFIs

Scattered cases of fusariosis have been reported in COVID-19 patients across the globe. For instance, two patients with severe COVID-19 infected with *F*. *verticillioides* and *F*. *proliferatum* were reported from Argentina and France, respectively [123, 124]. In addition, two cases of fusariosis were detected in mechanically-ventilated COVID-19 patients among subjects of observational studies from France and Spain [125, 126]. In one of the studies we reviewed, six COVID-19 patients with acute respiratory distress syndrome (ARDS) had pulmonary fusariosis [16].

It is noteworthy that none of these patients had the classic risk factors of invasive fusariosis, i.e., hematologic malignancy and history of transplant [127]. However, all of them were mechanically ventilated and received corticosteroids and antibiotics, emphasizing the role of COVID-19 and its treatment in triggering *Fusarium* invasion. Treatment of fusariosis is difficult due to the resistance to most antifungal drugs, its optimal treatment is yet to be discovered, and its mortality rate remains high [128–130]. Thus, it is not unexpected that all Iranian patients died, with this in mind that all were high-risk COVID-19 ICU patients and did not receive any antifungal drugs.

COVID-19 patients are prone to infections by uncommon fungal pathogens. Three instances of very rare fungal infections were reported in Iranian COVID-19 patients, namely *R*. *mucilaginosa* and *S*. *kiliense*, and *D*. *foeniculina* [16, 19, 31]. This highlights the importance of considering emerging and rare fungi as a differential diagnosis of COVID-19 complications. To the best of our knowledge, these fungi have not been reported in other patients with COVID-19 at the time of writing this article.

Eleven cases described as probable pulmonary mold infections were detected by serum or BAL GM [16]. GM testing in serum and BAL samples is used as a screening and diagnostic tool; however, its positive predictive value ranges between 0 and 100% [131]. Therefore, the diagnosis of mold infection in these cases is undetermined, as the respiratory features and radiological findings can be attributed to SARS-CoV-2 or other organisms.

## Conclusion

Fungal infections are evident to be a complication of COVID-19 in Iran, especially in patients with diabetes mellitus, cardiovascular diseases, and those receiving antibiotics, corticosteroids, and mechanical ventilation. Candidiasis, mucormycosis and aspergillosis are the most common CAFIs in Iran. However, less common infections like fusariosis and cases of rare infections have also been reported. Accordingly, clinicians should avoid neglecting fungal infections by acknowledging them as differential diagnoses and ordering confirmative laboratory tests in high-risk patients or those not responding to routine treatments.

## Supporting information

S1 File PRISMA checklist. (DOCX)

S2 File PubMed search strategy. (DOCX)

S3 File Risk of bias assessment results. (XLSX)

S4 File Extracted data. (XLSX)
