# Supplementary material for: COVID-19-associated fungal infections in Iran: A systematic review
Source: PLoS One. 2022 Jul 11;17(7):e0271333. doi: 10.1371/journal.pone.0271333 (PMC9273100; doi:10.1371/journal.pone.0271333)
Supplement: S2 File — (DOCX) [file pone.0271333.s002.docx]

**PubMed search strategy:**

((((((((((((((((((((((((((((mycoses[MeSH Terms]) OR (fungi[MeSH Terms])) OR (invasive fungal infections[MeSH Terms])) OR (candidiasis[MeSH Terms])) OR (candidiasis, vulvovaginal[MeSH Terms])) OR (candidiasis, cutaneous[MeSH Terms])) OR (candidiasis, invasive[MeSH Terms])) OR (candidiasis, oral[MeSH Terms])) OR (aspergillosis[MeSH Terms])) OR (pulmonary aspergillosis[MeSH Terms])) OR (invasive pulmonary aspergillosis[MeSH Terms])) OR (neuroaspergillosis[MeSH Terms])) OR (mucormycosis[MeSH Terms])) OR (cryptococcus[MeSH Terms])) OR (carinii, pneumocystis[MeSH Terms])) OR (candida[MeSH Terms])) OR (aspergillus[MeSH Terms])) OR (mucor[MeSH Terms])) OR (rhizopus[MeSH Terms])) OR (fung*[Title/Abstract])) OR (mycos*[Title/Abstract])) OR (candid*[Title/Abstract])) OR (mucor*[Title/Abstract])) OR (aspergill*[Title/Abstract])) OR (cryptococc*[Title/Abstract])) OR (pneumocyst*[Title/Abstract])) OR (Zygomycosis[MeSH Terms])) OR (zygomyc*[Title/Abstract])) OR (phycomyc*[Title/Abstract])

AND

((((((((((((((COVID-19[MeSH Terms]) OR (SARS-CoV-2[MeSH Terms])) OR (SARS-CoV-2 variants [Supplementary Concept])) OR (COVID-19[Title/Abstract])) OR ("COVID 19"[Title/Abstract])) OR (2019-nCoV[Title/Abstract])) OR (Coronavirus[Title/Abstract])) OR ("Novel Coronavirus"[Title/Abstract])) OR ("2019 nCoV"[Title/Abstract])) OR (COVID19[Title/Abstract])) OR (SARS-CoV-2[Title/Abstract])) OR ("SARS CoV 2"[Title/Abstract])) OR ("Severe Acute Respiratory Syndrome Coronavirus 2"[Title/Abstract])) OR ("Wuhan Coronavirus"[Title/Abstract])) OR (SARSCoV2[Title/Abstract])

AND

((((((iran[Affiliation]) OR (iran[Author - Corporate])) OR (iran[Title/Abstract])) OR (iran[MeSH Terms])) OR (iran[Other Term])) OR (iran[Grant Number])) OR (iran[PL])

AND

"2020"[Date - Publication] : "2021"[Date - Publication]

AND

"english"[Language]
